# DNA methylation signatures support the role of neutrophils and monocytes in depression

**DOI:** 10.1192/j.eurpsy.2024.542

**Published:** 2024-08-27

**Authors:** E. Atil, E. Van Assche, C. Hohoff, B. T. Baune

**Affiliations:** ^1^University of Münster; ^2^University of Münsterr, Münster, Germany

## Abstract

**Introduction:**

Research repeatedly linked inflammation with major depressive disorder (MDD). The presence of an inflammatory subtype of depression is supported by molecular findings as well as imaging reports. We investigated the cell type composition estimated by using epigenome-wide DNA methylation markers in a sample of depressed individuals showing high or low inflammation levels measured by hsCRP. We aimed to understand the connection between depression and inflammation, specifically differences in cell type compositions between high and low inflammation groups at baseline.

**Objectives:**

119 individuals with MDD were included for this analysis. Following quality control procedures, 113 participants were included in the analysis (M_age_= 47 years, 57.98% women). The sample consisted of 37 individuals with high hsCRP (hsCRP > 1.5, M_age_=45, M_hsCRP_=8.2, M_MADRS_=28, 70% women) and 76 individuals with low hsCRP (hsCRP < 1.5, M_age_= 44, M_hsCRP_=0.99, M_MADRS_=28, 49% women).

**Methods:**

The Illumina Infinium MethylationEPIC 850k BeadChip was used for analyzing whole blood derived DNA. Data processing and cell type estimation was conducted using the RnBeads package. We applied the Houseman method to estimate cell type composition through epigenome-wide DNA methylation signatures, resulting in six cell types: neutrophils, natural killer cells, B cells, CD4+ T cells, CD8+ T cells and monocytes. Comparisons between both groups were tested using ANOVA.

**Results:**

High and low hsCRP groups were compared for each of the six cell types estimated. A statistically significant difference was seen for monocytes (p=0.0316) and a trend for neutrophils (p=0.0742). The mean values for neutrophils in patients without inflammation were found to be 60%, while in patients with inflammation, it was 63%. For monocytes, the mean values for patients without inflammation and those with inflammation were 10% and 9.4%, respectively, with a smaller range (4.5%-14.3%) for individuals with inflammation as compared to patients without inflammation (5.3%-20.7%). None of the other four cell types showed a statistically significant difference.

**Conclusions:**

We identified differences in the cell type composition between groups of depressed patients with high versus low inflammation. These results align with the existing body of knowledge reported in established academic literature. Our study emphasizes the role of specific cells like neutrophils and monocytes in inflammation and depression. These findings offer valuable insights for improving depression treatment strategies as inflammation state may be relevant for treatment response. We also show the merit of DNA methylation signatures for the profiling of patients’ inflammation status, i.e., immunomethylomics.

**Disclosure of Interest:**

None Declared

